# Data Mining and Network Pharmacology Analysis of Kidney-Tonifying Herbs on the Treatment of Renal Osteodystrophy Based on the Theory of “Kidney Governing Bones” in Traditional Chinese Medicine

**DOI:** 10.1155/2022/1116923

**Published:** 2022-09-30

**Authors:** Xue Tong, Yuanjian Yang, Shuwei Gong, Xiao Han, Yanfei Wu, Xiaoran Ma, Shuang Lang, Jianxiong Ma, Xinlong Ma

**Affiliations:** ^1^School of Integrative Medicine, Tianjin University of Traditional Chinese Medicine, Tianjin, China; ^2^Tianjin Key Laboratory of Orthopedic Biomechanics and Medical Engineering, Orthopedic Research Institute, Tianjin Hospital, Tianjin, China; ^3^Tianjin Hospital, Tianjin, China; ^4^Tianjin Jinnan Traditional Chinese Medicine Hospital, Tianjin, China

## Abstract

**Background:**

Renal osteodystrophy (ROD) secondary to chronic kidney disease is closely associated with osteoporosis and fractures. Based on the theory of “kidney governing bones” in traditional Chinese medicine (TCM), treating bone diseases from the perspective of the kidney has become a basic principle of treating ROD. However, there are many kidney-tonifying herbs and their mechanisms of treating ROD are not clear. Therefore, our study intends to use data mining and network pharmacology to study the commonly used kidney-tonifying herbs, as well as their active ingredients and mechanisms of treating ROD.

**Methods:**

We established a clinical ROD database by searching PubMed, CNKI, and other databases and screened out a core herbal combination of treating ROD. Furthermore, by using databases such as Traditional Chinese Medicine Systems Pharmacology Database and Analysis Platform and GeneCards, we obtained active ingredients and targets of the core herbal combination and ROD targets. The STRING website and Cytoscape software were then used to obtain information on key active ingredients and key targets. Finally, we conducted GO and KEGG analyses using the Metascape website and molecular docking using the AutoDock Vina software.

**Results:**

Our study eventually included 58 prescriptions and 116 herbs of treating ROD. Through data mining, we found that yin-yang-huo, du-zhong, and bu-gu-zhi (YDB) constituted a core herbal combination to treat ROD. Network pharmacology showed that YDB mainly acted on targets such as estrogen receptor alpha through active ingredients such as quercetin by mitogen-activated protein kinase and other signaling pathways.

**Conclusion:**

Many ingredients, targets, and pathways are involved in the treatment of YDB for ROD. Specifically, the flavonoids contained in YDB have great potential for ROD treatment.

## 1. Introduction

In 2005, Kidney Disease: Improving Global Outcomes (KDIGO) proposed the concept of chronic kidney disease-mineral and bone disorder (CKD-MBD), which was intended to emphasize the systemic mineral and bone metabolic disorders caused by CKD [1]. CKD-MBD manifests as abnormalities in mineral and bone metabolism and/or extra-skeletal calcification; while the original term “renal osteodystrophy” (ROD) specifically refers to CKD-related bone histological abnormalities, including abnormalities in bone transformation, bone mineralization, bone mass, or bone strength [1, 2].

ROD, which is very common in CKD patients, has already occurred in the early stage of CKD [3]. As the glomerular filtration rate (GFR) declines, bone remodeling and microstructure become progressively worse. In stage 5 CKD, almost all patients have abnormal bone performance [3]. A survey showed that the prevalence of osteoporosis in people with estimated-GFR less than 60 mL/min was twice higher than those with estimated-GFR greater than 60 mL/min [4]. According to the rate of bone turnover, ROD can be divided into high-turnover ROD, low-turnover ROD (including adynamic bone disease and osteomalacia), and mixed lesions [2, 5]. However, regardless of the type of bone turnover, ROD can lead to increased fracture risk and cardiovascular diseases and is associated with premature death [4, 6].

The theory of “kidney governing bones” originated in the Yellow Emperor's Classic of Internal Medicine (“hangdi neijing” in Chinese), which suggests that “treatment from the kidney” is an important idea for treating bone diseases [7]. The ancients put forward the theory of “kidney storing essence, essence producing marrow, and marrow filling bones” through the discovery of anatomy and long-term observation of their physiological activities, which was then simplified as the “kidney governing bones” theory [8]. TCM believes that kidney deficiency and injured bone (“shenxu gusun” in Chinese) and turbidity and blood stasis (“zhuoyu hujie” in Chinese) are the basic pathogenesis of ROD. Correspondingly, the treatment methods aim to tonify the kidney and strengthen the bone (“bushen zhuanggu” in Chinese), tonify the kidney and strengthen the spleen (“bushen jianpi” in Chinese), and tonify the kidney and activate blood circulation (“bushen huoxue” in Chinese) [7]. With the discoveries of molecules and their roles in the regulation of kidneys and bones such as bone morphogenetic protein (BMP), parathyroid hormone (PTH), fibroblast growth factor 23 (FGF23), and klotho protein, modern medicine has increasingly recognized that the relationship between kidneys and bones is very close, which further expands and extends the theory of “kidney governing bones” in TCM [9]. In addition, these molecules are also essential in the pathogenesis of ROD [10].

By using modern computing technology, data mining can analyze medication patterns from clinical prescription data, so as to find potential relationships between herbs and diseases and between different herbs, which is an important embodiment of TCM informatization [11].

**Figure 1:**
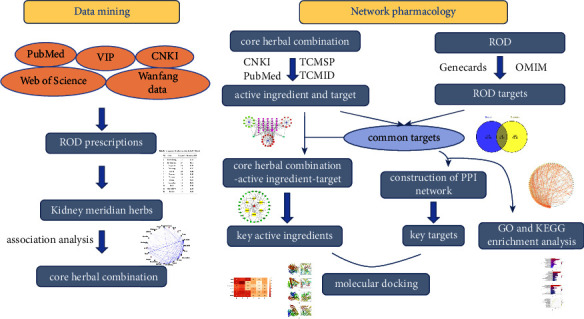
The workflow of our study.

Based on the “multi-ingredient and multitarget” nature of herbs and the multigene pathogenesis of disease, network pharmacology reveals the complex bioinformatics network of herb-ingredient-target-disease through high-throughput screening and can predict the potential mechanisms of herbs [12–17]. Therefore, data mining combined with network pharmacology can explain the medication rules of ROD, especially kidney-tonifying herbs, with pharmacological mechanisms on the basis of clinical database, which provides more ideas for basic research and treatments of ROD. The workflow of our study is illustrated in Figure 1.

## 2. Materials and Methods

### 2.1. Prescription Source

Our prescriptions were obtained from databases including PubMed (https://pubmed.ncbi.nlm.nih.gov/), Web of Science (https://webofscience.com), CNKI (https://www.cnki.net/), Wanfang data (https://www.wanfangdata.com.cn/index.html), and VIP database (https://www.cqvip.com/). Based on these databases, we conducted a systematic search using two sets of keywords. The first set of keywords included “ROD” OR “renal bone disease” OR “high turnover bone disease” OR “low turnover bone disease” OR “adynamic bone disease” OR “osteomalacia”. The second set of keywords included (“CKD” OR “chronic renal failure” OR “uremia” OR “hemodialysis” OR “peritoneal dialysis”) AND (“bone abnormalities” OR “bone mineral density (BMD)” OR “bone biopsy” OR “bone metabolism” OR “bone remodeling” OR “bone pain”). Then, we screened and identified all relevant studies with TCM prescriptions.

### 2.2. Inclusion and Exclusion Criteria

#### 2.2.1. Inclusion Criteria

Clinical studies using TCM prescriptions or proprietary Chinese medicine with clear herbal composition and curative effects, such as randomized controlled trials, cases of TCM experts (only the first prescription was included), and case report studies were included. Studies with same prescriptions were included only once.

#### 2.2.2. Exclusion Criteria

Animal or cell experimental studies, purely theoretical studies, studies with unknown prescription or single herb, and studies on the treatment of acupuncture and external use of TCM were excluded.

### 2.3. Selection and Standardization of Kidney-Tonifying Herbs

Kidney-tonifying herbs are mostly kidney meridian herbs, and its definition is vague in clinical practice. Therefore, we expanded the search scope and extracted kidney meridian herbs by referring to “Chinese Pharmacopoeia” (2020 Edition) and “Chinese Materia Medica” in the prescriptions of treating ROD; additionally, standardizing their names based on these two books, for example, standardizing “salt du-zhong” as “du-zhong”.

### 2.4. Association Analysis

The apriori algorithm was used in SPSS Modeler 18.0 software to conduct an association analysis on kidney meridian herbs [18]. According to “the support ≥ 15%, the confidence ≥ 85%, and the maximum number of antecedents was 2,” the core herbal combination of treating ROD with TCM was obtained.

### 2.5. Network Pharmacology Research

#### 2.5.1. Acquisition of Active Ingredients and Targets of the Core Herbal Combination

First, the Traditional Chinese Medicine Systems Pharmacology Database and Analysis Platform (TCMSP, https://old.tcmsp-e.com/tcmsp.php) database was used to search active ingredients of the core herbal combination [19]. For herbs that can be found in the TCMSP database, according to ADME principles of “oral bioavailability ≥ 30% and drug-likeness (DL) ≥ 0.18,” we screened out active ingredients and protein targets [20]. Then, we used the UniProt website (https://www.uniprot.org/) [21] to match protein targets and gene names.

For herbs that were not included in the TCMSP database, their ingredients can be obtained from the Traditional Chinese Medicine Integrated Database (TCMID, https://www.megabionet.org/tcmid/) [22], CNKI, and PubMed databases. The obtained ingredients were then screened on the SWISS ADME website (https://www.swissadme.ch/) [23], and we selected ingredients with high gastrointestinal absorption and with at least 2 items of DL (lipinski, ghose, veber, egan, and muegge) being yes [24]. Next, the protein targets and standard gene names of screened ingredients were obtained from the SWISS TARGET PREDICTION website (https://www.swisstargetprediction.ch/) [25]. At last, by removing the repeated ingredients and targets obtained from the above two steps, the final active ingredients and protein targets can be obtained.

#### 2.5.2. Acquisition of ROD Targets

We searched the GeneCards database (https://www.genecards.org/) [26] and the OMIM database (https://omim.org/) [27] for ROD targets, and the final targets were obtained after merging and removing duplicate targets.

#### 2.5.3. Construction of Core Herbal Combination-Active Ingredient-Target Network

We obtained the common targets that were included in both herb targets and disease targets by taking the intersection and found the ingredients containing these common targets. Cytoscape 3.7.1 software was then used to construct the network of these active ingredients and targets [28]. Furthermore, based on the topological parameters, information of key active ingredients was obtained.

#### 2.5.4. Construction of Protein-Protein Interaction (PPI) Network

First, we expanded targets by importing common targets into the SRTING 11.5 website (https://cn.string-db.org/) [29], set the species to “*Homo sapiens*” and the item of “the max number of interactors to show” to “1st shell no more than 20 interactors and 2nd shell no more than 20 interactors”, and hid disconnected nodes in this network. Then, we saved the results in a TSV file and imported it into the Cytoscape 3.7.1 software to construct a PPI network diagram of the targets of YDB in treating ROD. Information about key targets can be obtained by screening topological parameters.

#### 2.5.5. Gene Ontology (GO) and Kyoto Encyclopedia of Genes and Genomes (KEGG) Enrichment Analyses

GO and KEGG enrichment analyses were performed on targets obtained in section 2.5.4 using Metascape website (https://metascape.org/) [30]. GO analysis is mainly used to describe gene functions, which can be divided into three aspects: cellular component (CC), biological process (BP), and molecular function (MF). KEGG enrichment analysis can obtain the signal pathways of core herbal combination in the treatment of ROD. The above results were visualized by https://www.bioinformatics.com.cn/, an online platform for data analysis and visualization.

#### 2.5.6. Molecular Docking Verification

Molecular docking verification was based on key active ingredients and key targets obtained under sections 2.5.3 and 2.5.4. First, we used the PubChem database (https://pubchem.ncbi.nlm.nih.gov/) to obtain 2D structures of key active ingredients. We then converted them to 3D structures, minimized their energy, and saved them in mol2 formats by using ChemBio 3D Ultra software [31]. Next, they were imported into AutoDockTools 1.5.7 software [32] and converted into the pdbqt format to complete the preparation of ligands. For preparation of receptors, the RCSB PDB database (https://www.rcsb.org/) was used to download protein structures of the key targets [33]. Next, these structures were imported into PyMOL software [32] to remove solvents and organic residues and then hydrotreated in AutoDockTools 1.5.7 software, the results of which were saved in a pdbqt file to complete the receptor preparation. Meanwhile, the active pockets of these structures were determined. Molecular docking and calculation of the lowest binding energy were conducted using AutoDock Vina 1.1.2 software [34], and docking modes were visualized by using PyMOL software.

## 3. Results and Discussion

### 3.1. The Frequency of Kidney Meridian Herbs

In 56 studies, 58 prescriptions containing 116 Chinese herbs were included in our study. These herbs have been used for a total 613 times in different prescriptions. Among these 116 herbs, there were 60 kidney meridian herbs (accounting for 51.72% of all herbs), which have been used for a total of 317 times (accounting for 51.71% of the total frequency). In addition, among the 60 kidney meridian herbs, there were 12 herbs with frequencies more than 10 times, which were shu-di-huang, yin-yang-huo, bu-gu-zhi, du-zhong, niu-xi, shan-yao, etc. Their frequencies are shown in Table 1, and their corresponding Latin names are shown in Supplementary Table S1. They were basically common kidney-tonifying herbs. To sum up, it can be seen that the application of kidney-tonifying herbs was very extensive in the treatment of ROD.

### 3.2. Prescription Rules Based on Association Analysis

**Figure 2:**
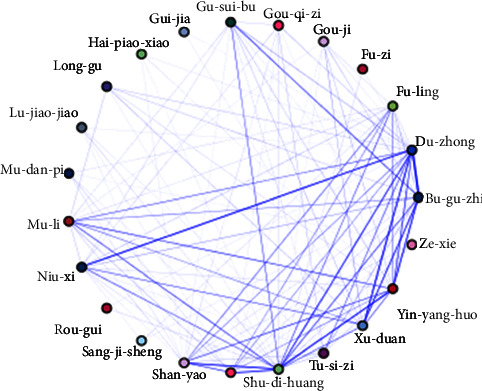
Association network diagram of high-frequency kidney meridian herbs. The darker the line, the higher the frequency of the two drugs appearing at the same time.

Two groups of herbal combinations were obtained under section 2.4, as shown in Table 2. According to the ranking of “the support” and “the confidence”, yin-yang-huo, du-zhong, and bu-gu-zhi (YDB), which were all common kidney-tonifying herbs, were selected as the core herbal combination for the treatment of ROD.  Figure 2 shows the association network of high-frequency kidney meridian herbs.

### 3.3. Acquisition of Active Ingredients and Targets of the Core Herbal Combination

By searching the TCMSP database, we only found information about du-zhong and yin-yang-huo. According to section 2.5.1., different search strategies were carried out.

1. By using the TCMSP database, 28 active ingredients and 529 protein targets of du-zhong and 23 active ingredients and 508 protein targets of yin-yang-huo were obtained. After deduplication, 47 active ingredients and 242 protein targets were remained, of which 229 protein targets were matched as gene names using the UniProt website.
2. By using TCMID and referring to CNKI and PubMed databases, a total of 40 active ingredients of bu-gu-zhi were obtained. According to the steps in 2.5.1. and screening out targets with “probability ≥ 0.100”, 24 active ingredients and 266 gene names of protein targets were obtained. Then, ingredients and targets obtained in the previous steps were combined and deduplicated, and 71 active ingredients and 424 protein targets with gene names were remained for the treatment of ROD with YDB.

### 3.4. Acquisition of ROD Targets

We obtained 201 targets from the OMIM database and 157 targets from the GeneCards database, and a total of 354 targets were remained after deduplication.

### 3.5. Construction of YDB-ActiveIngredient-Target Network

**Figure 3:**
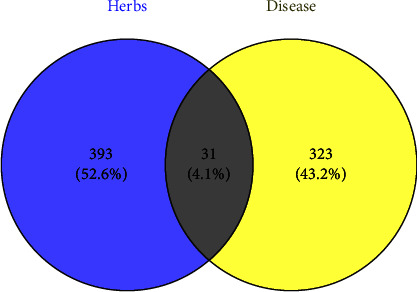
Venn diagram of herbs and disease targets.

**Figure 4:**
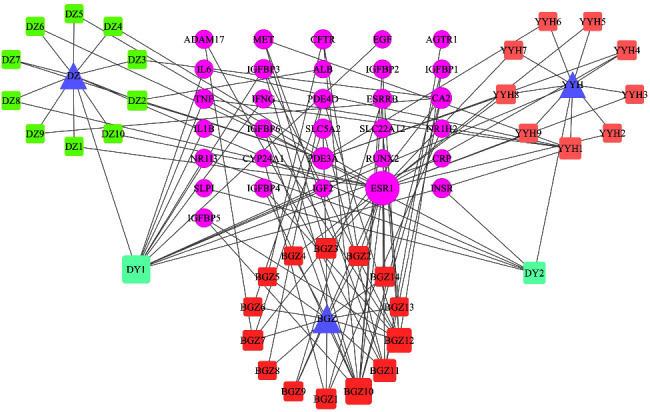
Core herb combination-activeingredient-target network of YDB in treating ROD. Herbs are represented by triangles, in which DZ stands for du-zhong, YYH stands for yin-yang-huo, and BGZ stands for bu-gu-zhi. Active ingredients are represented by round rectangles. Among them, DY1 and DY2 are the common ingredients of DZ and YYH. Targets are indicated by ellipses. The larger the node, the higher the degree value. The full names of abbreviations are shown in Supplementary Table S2.

**Figure 5:**
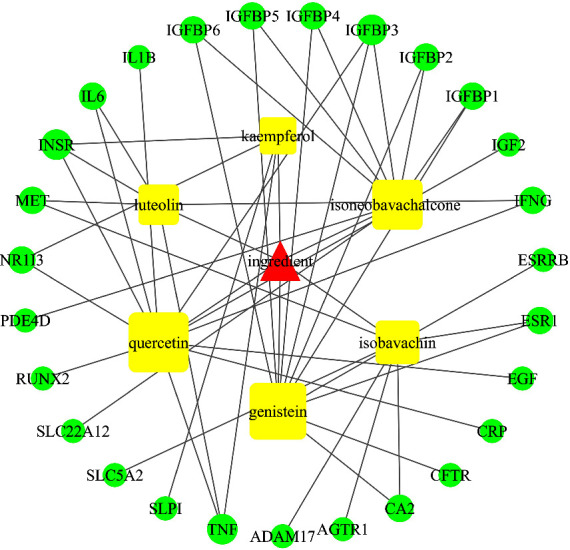
Key active ingredient-target network of YDB in treating ROD. Key active ingredients are represented by round rectangles. Targets are indicated by ellipses. The larger the node, the higher the degree value.

We obtained 31 common targets that were contained in both 424 herbal targets and 354 disease targets, which were considered to be the potential targets of YDB in treating ROD.  Figure 3 presents the Venn diagram conducted using the Venny 2.1 online website [35]. Then, 35 active ingredients were found to contain these targets, and Cytoscape 3.7.1 was used to construct the YDB-activeingredient-target network diagram, as shown in Figure 4. It can be seen that YDB, active ingredients, and targets were interconnected through a complex network, which consisted of 69 nodes and 122 edges. Each ingredient corresponded to multiple targets, reflecting the “multi-ingredient and multitarget” characteristic of YDB in treating ROD. The larger the node, the higher the degree value, indicating that the active ingredients had more potential targets. According to the network topology parameters, active ingredients with “degree > twice the median (4)” were screened out, namely, quercetin, genistein, isobavachin, luteolin, and kaempferol, as shown in Table 3. These ingredients were considered to be the key active ingredients of YDB in treating ROD, and the network diagram of key active ingredients and their targets is shown in Figure 5.

### 3.6. Construction of PPI Network

**Figure 6:**
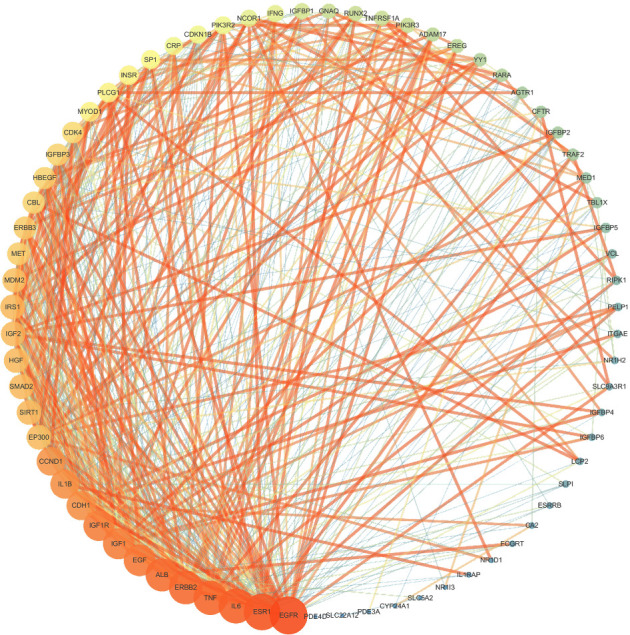
PPI network of YDB in treating ROD.

According to section 2.5.4 for targets expansion, we obtained a network with 71 targets and 593 edges, and the average node degree was 16.7. The data of this network were then imported into Cytoscape 3.7.1 software to obtain the PPI network of YDB in treating ROD, as shown in Figure 6. We found that the larger and brighter the node, the greater the degree value. In addition, the thicker and brighter the edge, the greater the combined-score value.

Degree, betweenness centrality, and closeness centrality are all network topology parameters used to measure the importance and centrality of nodes [36, 37]. The larger the degree value, the more the number of connects of a node. The betweenness centrality refers to the ratio of the shortest pathways passing through a node to all the shortest pathways between any two nodes in the network, and the closeness centrality reflects the closeness of a node to other nodes in the network [36, 37]. Based on “degree > twice the median (26), betweenness centrality > median (0.00452157), and closeness centrality > median (0.51094891),” a total of 12 targets were screened out. These targets were then intersected with 31 common targets, and finally 6 targets were remained. These 6 targets may play a key role in the treatment of ROD and were considered to be the key targets of YDB in treating ROD. The 6 targets were ESR1, IL6, TNF, ALB, EGF, and IL1B, as shown in Table 4.

### 3.7. GO and KEGG Pathway Enrichment Analyses

**Figure 7:**
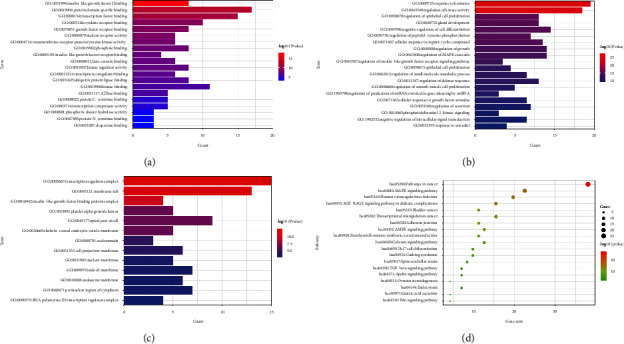
GO and KEGG pathway enrichment analyses of YDB in treating ROD: (a) MF analysis, (b) BP analysis, (c) CC analysis, and (d) KEGG pathway analysis.

The 71 targets were imported into the Metascape website for GO and KEGG pathway enrichment analyses, respectively. GO enrichment analysis of YDB in the treatment of ROD showed that there were 74 items in MF, 854 items in BP, and 51 items in CC.  Figure 7 presents the top 20 items sorted by the −lgP value in MF, BP, and CC, respectively. It can be seen that MF mainly enriched in insulin-like growth factor-1(IGF-1) binding, protein domain-specific binding, transcription factor binding, cytokine receptor binding, growth factor receptor binding, nuclear receptor activity, and *β*-catenin binding, among which protein domain-specific binding enriched the most genes. BP mainly focused on response to hormone, regulation of kinase activity, regulation of epithelial cell proliferation, cellular response to organic cyclic compounds, and regulation of mitogen-activated protein kinase (MAPK) cascade. There were 13 clustering results of CC, which mainly concentrated in transcription regulator complex, membrane raft, apical part of cell, clathrin-coated endocytic vesicle membrane, cell projection membrane, and nuclear membrane.

The pathways for YDB to treat ROD mainly enriched in 157 pathways, and there were 19 clusters in total after the hierarchical cluster method, ranked by the −lgP value from the largest tothe smallest, as shown in Figure 7(d). It can be seen that the molecular pathways mainly concentrated in the MAPK signaling pathway, 5′-adenosine monophosphate (AMP)-activated protein kinase (AMPK) signaling pathway, PTH synthesis, secretion, and action, calcium signaling pathway, transforming growth factor-*β* (TGF-*β*) signaling pathway, ovarian steroidogenesis, and Wnt signaling pathway.

### 3.8. Molecular Docking Verification

**Figure 8:**
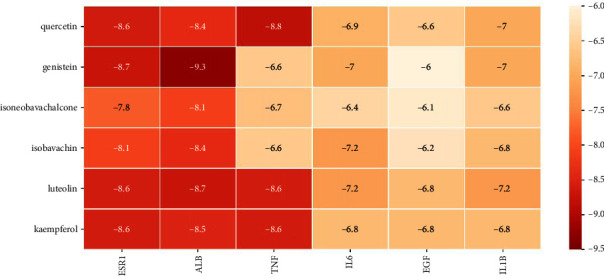
Molecular docking results of key active ingredients and key targets (kcal mol^−1^). The PDB IDs of ESR1, ALB, TNF, IL6, EGF, and IL1B are 7RS8, 7DJN, 1A8M, 1ALU, 1JL9, and 6Y8I, respectively.

**Figure 9:**
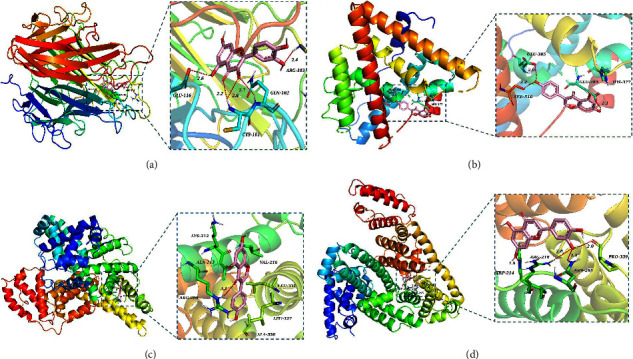
Molecular docking modes of some key active ingredients and key targets: (a) quercetin-TNF (PDB ID: 1A8M), (b) genistein-ESR1 (PDB ID: 7RS8), (c) genistein-ALB (PDB ID: 7DJN), and (d) luteolin-ALB (PDB ID: 7DJN).

We conducted molecular docking verification of 6 key active ingredients and 6 key targets, and a total of 36 results were obtained, which were expressed by the lowest binding energies (kcal·mol^−1^), as shown in Figure 8. It is generally believed that the more stable the ligand-receptor binding conformation, the lower the binding energy, and the binding energy < −4.25 kcal mol^−1^ can be considered to have the binding activity. Binding energy < −5.0 kcal mol^−1^ indicates a good binding activity, and binding energy < −7.0 kcal mol^−1^ indicates a strong binding activity [18, 38]. It can be calculated from Figure 8 that the average binding energy of key ingredients and key targets was −7.5 kcal mol^−1^, indicating that these ingredients and targets generally had strong binding activities. Among them, the targets with higher binding affinities were concentrated in ESR1, TNF, and ALB and the highest binding affinities were genistein and ALB, with the binding energy of −9.3 kcal mol^−1^, followed by quercetin and TNF (−8.8 kcal mol^−1^), genistein and ESR1 (−8.7 kcal mol^−1^), and luteolin and ALB (−8.7 kcal mol^−1^). The binding modes are shown in Figure 9.

## 4. Discussion

Essentially, ROD is the abnormal bone remodeling caused by CKD [39]. Bone remodeling maintains the normal structures and functions of bones mainly by balancing the bone formation of osteoblasts and the bone resorption of osteoclasts. When the balance of bone formation and bone resorption is disrupted, the structures and qualities of bones will change, and abnormal bone remodeling will occur [40]. By searching different databases and the association analysis based on the apriori algorithm, we concluded that YDB is the core herbal combination for treating ROD in TCM.

Du-zhong, yin-yang-huo, and bu-gu-zhi are all common kidney-tonifying herbs. Du-zhong and yin-yang-huo can strengthen bones directly or by tonifying the kidney [41, 42]. Bu-gu-zhi can tonify kidney yang and treat ROD with deficiency of kidney yang as the main symptoms [43]. From the perspective of modern medicine, the significant kidney-tonifying effects of bu-gu-zhi and its antiosteoporosis effects are related to its strong estrogen-like activities [44]. Patients with ROD often have osteoporosis [45]. The study of Ha et al. showed that the components in a part of the fractions of du-zhong can participate in the proliferation, differentiation, and maturation of osteoblasts and can inhibit the activities of osteoclasts to play the role of antiosteoporosis [46, 47]. ROD is secondary to CKD, and the total flavonoids in Epimedium koreanum Nakai have been shown to alleviate chronic renal failure in rats by activating AMPK-related pathways [48]. Besides, yin-yang-huo can stimulate small extracellular vesicles in bone mesenchymal stem cells (MSCs) to secrete miR-27a-5p, targeting Atg4B to stimulate osteogenesis [49].

By taking the intersection of herbal targets and disease targets, we obtained the potential targets of YDB in treating ROD. Then, by constructing the network of core herbal combination-activeingredient-target and PPI network of expanded targets, the information about key active ingredients and key targets was obtained, respectively. The key active ingredients were quercetin, genistein, isoneobavachalcone, isobavachin, luteolin, and kaempferol, and the key targets were ESR1, IL6, TNF, ALB, EGF, and IL1B.

Quercetin, genistein, luteolin, and kaempferol all belong to flavonoids, which have been developed into nutraceuticals due to the abilities of anti-inflammatory, improving oxidative stress, and regulating the activities of key cellular enzymes [50]. Flavonoids also involve in multiple pathways that regulate bone remodeling and have the great potential in the treatment of bone diseases [51]. Quercetin is a flavonol. Through the summary of a large number of animal experiments, Wong et al. found that quercetin can regulate various molecules and pathways of bone remodeling to promote bone formation and inhibit bone resorption [52]. Yang et al. found that quercetin can significantly reduce the levels of inorganic phosphorus, FGF23, and PTH in an adenine-induced CKD rat model, indicating that quercetin has the function of regulating FGF23 and PTH, which are crucial factors to the regulation of blood phosphorus and the occurrence of ROD [53]. Receptor activator of nuclear factor-*κ*B ligand (RANKL) is a key molecule that promotes bone resorption [54]. Osteoprotegerin (OPG) is a receptor decoy for RANKL, which can block the binding of RANKL to its receptor RANK, so as to inhibit bone resorption [54]. Genistein is derived from bu-gu-zhi and is a phytoestrogen [51]. The study of Li et al. showed that low-dose genistein can upregulate the expression of bone-specific alkaline phosphatase (ALP), OPG, and osteocalcin (OCN), and downregulate the expression of RANKL through the mediation of estrogen receptor beta (ESR2), suggesting that genistein can promote bone formation and inhibit bone resorption [55]. Luteolin and kaempferol were also shown to block RANKL-induced differentiation of RAW264.7 cells to osteoclasts, as well as reduce the generation of inflammatory mediators such as IL6, TNF-*α*, and IL1, which play a crucial role in enhancing RANKL expression and stimulating osteoclast activation [51, 56–58]. Our molecular docking results also verify the above view, showing that the binding energies of quercetin, luteolin, and kaempferol with TNF, IL6, and IL1B were all less than −5 kcal mol^−1^, and the docking abilities with TNF were the strongest. Isoneobavachalcone and isobavachin are components of bu-gu-zhi, which can also affect bone remodeling. The study of Lee et al. showed that isobavachin can inhibit RANKL signaling, and isoneobavachalcone can affect the activity of cathepsin K (CTSK), a specific protease secreted by osteoclasts and responsible for the degradation of collagen type I in the bone matrix [59].

Besides TNF, IL6, and IL1B, the rest three targets including EGF, ESR1, and ALB also play an important role in regulating bone remodeling. Specifically, EGF can promote the proliferation of osteoblasts and osteoclasts [60]. However, its effect on osteoblast differentiation can be either promotive or prohibitive depending on the differences in the experimental settings [60]. ESR1 is another receptor of estrogen, which is distributed in osteocytes, osteoblasts, and osteoclasts [61]. Our docking results showed that ESR1 had the strongest binding activity with genistein among all key components, which is as expected because genistein belongs to phytoestrogen. The pro-osteogenic effects of estrogen manifest that it can promote the proliferation and differentiation of MSCs into osteoblasts, increase the expression of OPG, promote the apoptosis of osteoclasts, and inhibit the expression of osteoclast factors [61, 62]. The study of Wan et al. showed that estrogen levels were low in ROD hemodialysis patients of childbearing age and negatively correlated with PTH [63]. ALB is more related to CKD. Kovesdy and Kalantar-Zadeh showed that ALB is still the strongest predictor of survival in patients with CKD [64]. In addition, plant polyphenols such as flavonoids exert biological activities by binding to serum ALB, which is in line with the results of our molecular docking: the key active components all having strong binding activities to ALB [65]. Therefore, it can be speculated that the low serum ALB levels in CKD/ROD patients limit the abilities of these compounds to modulate bone remodeling.

As mentioned earlier, ROD is actually the abnormal bone remodeling caused by CKD [39]. The process of bone remodeling is very complicated, involving the participation of various molecules and signaling pathways. By importing the expanded targets into the Metascape website, we obtained several major signaling pathways such as MAPK pathway and TGF-*β* signaling pathway. These pathways are ways for the therapeutic effects of kidney-tonifying herbs and also suggest the pathogenesis of ROD.

(1) MAPK signaling pathway: it involves in the biological process of osteoblasts and osteoclasts from development to maturity, and the transmission of multiple molecule signals [66, 67]. Both estrogen and inflammatory mediators such as TNF-*α*, IL6, and IL1B can regulate bone remodeling by activating the MAPK signaling pathway [58, 62, 68]. (2) PTH synthesis, secretion, and action: PTH is a vital hormone for the connection of kidneys and bones. The abnormal metabolism of calcium, phosphorus, and vitamin D caused by CKD can bring about changes in PTH levels and then affect normal bone metabolism and bone remodeling [10]. Moreover, PTH coordinates various factors and pathways that regulate bone remodeling, such as MAPK, TGF-*β*, BMP, wingless related MMTV integration site (Wnt), FGF23, and IGF-1 [69]. Therefore, the kidney-PTH pathway is considered to be the axis pathway in the pathogenesis of ROD [10]. (3) TGF-*β*/BMP signaling pathway: TGF-*β*/BMP belongs to the TGF-*β* superfamily and also plays crucial roles in bone remodeling [70, 71]. The effects of TGF-*β* on bone remodeling are complicated. In vivo, the interactions with other growth factors in the bone environment and the environment as such determine the final outcome of TGF-*β* on bone remodeling [72]. The major role of BMP is to promote osteogenesis [71]. Wu et al. found that, in ROD rats, indicators related to bone metabolism including phosphorus, intact PTH, and ALP were increased, while BMP2, BMD, and calcium were decreased [73]. After treatment with Shenshui Nutrition Capsule, a Chinese patent medicine, the above indicators were improved, so they believed that the mechanism of improving bone metabolism was related to the upregulation of BMP2 [73]. (4) Wnt signaling pathway: the Wnt pathway includes canonical Wnt pathway and noncanonical Wnt pathway [74]. The role of canonical Wnt pathway on bone formation is relatively clear and is *β*-catenin dependent [74]. Many herbs, including yin-yang-huo, du-zhong, and bu-gu-zhi can stimulate born formation through the Wnt/*β*-catenin signaling pathway [75, 76]. Besides regulating bone, Wnt/*β*-catenin is also involved in kidney damage and repair [77]. In many CKD models, upregulation of Wnt/*β*-catenin is found [77]. Therefore, the Wnt/*β*-catenin pathway is closely related to the pathogenesis of ROD.

## 5. Conclusion

By data mining, we concluded that YDB is the core herbal combination for ROD treatment. Furthermore, network pharmacology showed that the key targets of therapeutic effects were concentrated on ESR1, IL6, TNF, ALB, EGF, and IL1B, and the key components were concentrated on quercetin, genistein, isoneobavachalcone, isobavachin, luteolin, and kaempferol, which all belong to flavonoids, suggesting their great potential in the treatment of ROD. Therefore, we suggest that more research should be devoted to the effects and mechanisms of flavonoids on bones and kidneys under the circumstance of ROD or CKD-MBD in the future. The essence of ROD is the abnormal bone remodeling caused by CKD, which is regulated by a complicated network involving multiple molecules and pathways. Through KEGG pathway enrichment analysis, MAPK, PTH, TGF-*β*, Wnt, and other pathways were obtained in our study. In conclusion, our study reveals the “multicomponent, multitarget, and multipathway” effects of YDB in the treatment of ROD through data mining and network pharmacology based on the theory of “kidney governing bones” in TCM. Moreover, the potential roles of flavonoids in the treatment of ROD are worth further study.

## Figures and Tables

**Table 1:** The frequency of kidney meridian herbs (≥10 times).

No.	Herbs	Frequency	Percentage (%)
1	Shu-di-huang	27	46.55
2	Yin-yang-huo	27	46.55
3	Bu-gu-zhi	25	43.10
4	Du-zhong	25	43.10
5	Niu-xi	20	34.48
6	Shan-yao	20	34.48
7	Xu-duan	20	34.48
8	Fu-ling	18	31.03
9	Gu-sui-bu	16	27.59
10	Mu-li	16	27.59
11	Shan-zhu-yu	15	25.86
12	Tu-si-zi	11	19.00

**Table 2:** Herbal combinations for the treatment of ROD.

Herbal combination	Support (%)	Confidence (%)
Yin-yang-huo, du-zhong, bu-gu-zhi	18.966	90.909
Niu-xi, xu-duan, du-zhong	15.517	88.889

**Table 3:** Key active ingredients of YDB in treating ROD.

Herb	Abbreviation	Ingredient	PubChem CID	Degree value
Du-zhong/Yin-yang-huo	DY1	Quercetin	5280343	11
Bu-gu-zhi	BGZ10	Genistein	5280961	10
Bu-gu-zhi	BGZ12	Isoneobavachalcone	5318608	8
Bu-gu-zhi	BGZ11	Isobavachin	193679	6
Yin-yang-huo	YYH1	Luteolin	5280445	5
Du-zhong/Yin-yang-huo	DY2	Kaempferol	5280863	4

**Table 4:** Key targets information of YDB in treating ROD.

Gene target	Degree	Betweenness centrality	Closeness centrality
ESR1	45	0.09171257	0.70707071
IL6	42	0.06178373	0.68627451
TNF	41	0.06550194	0.67961165
ALB	39	0.10959957	0.67961165
EGF	37	0.01999811	0.64220183
IL1B	33	0.03606639	0.63063063

## Data Availability

The data used to support the findings of this study can be obtained from the corresponding author upon request.

## Supplementary Materials

Supplementary Materials Table S1: The Pinyin names and their corresponding Latin names (Supplementary Material 1). Table S2: The names of active ingredients represented by abbreviations (Supplementary Material 2).
