# Supplementary material for: Data Mining and Network Pharmacology Analysis of Kidney-Tonifying Herbs on the Treatment of Renal Osteodystrophy Based on the Theory of “Kidney Governing Bones” in Traditional Chinese Medicine
Source: Evid Based Complement Alternat Med. 2022 Sep 30;2022:1116923. doi: 10.1155/2022/1116923 (PMC9552684; doi:10.1155/2022/1116923)
Supplement: Supplementary Materials — Table S1: The Pinyin names and their corresponding Latin names (Supplementary Material 1). Table S2: The names of active ingredients represented by abbreviations (Supplementary Material 2). [file 1116923.f1.zip › 1116923.f1/Supplementary Material (1).doc]

**Supplementary Material:**

Table S1. The Pin-yin names and their corresponding Latin names

| Herb Pinyin | Latin Name |
| --- | --- |
| Shu-di-huang | *Rehmannia glutinosa (Gaertn.) DC.* |
| Yin-yang-huo | *Epimedium brevicornu Maxim. / Epimedium sagittatum (Siebold & Zucc.) Maxim. / Epimedium pubescens Maxim. / Epimedium koreanum Nakai* |
| Bu-gu-zhi | *Cullen corylifolium (L.) Medik.* |
| Du-zhong | *Eucommia ulmoides Oliv.* |
| Niu-xi | *Achyranthes bidentata Blume* |
| Shan-yao | *Dioscorea oppositifolia L.* |
| Xu-duan | *Dipsacus asper Wall. ex DC.* |
| Fu-ling | *Poria cocos (Schw.) Wolf* |
| Gu-sui-bu | *Drynaria fortunei (Kunze) J. Sm.* |
| Mu-li | *Ostrea gigas thunberg* |
| Shan-zhu-yu | *Cornus officinalis Siebold & Zucc.* |
| Tu-si-zi | *Cuscuta australis R.Br. / Cuscuta chinensis Lam.* |
