# Supplementary material for: Data Mining and Network Pharmacology Analysis of Kidney-Tonifying Herbs on the Treatment of Renal Osteodystrophy Based on the Theory of “Kidney Governing Bones” in Traditional Chinese Medicine
Source: Evid Based Complement Alternat Med. 2022 Sep 30;2022:1116923. doi: 10.1155/2022/1116923 (PMC9552684; doi:10.1155/2022/1116923)
Supplement: Supplementary Materials — Table S1: The Pinyin names and their corresponding Latin names (Supplementary Material 1). Table S2: The names of active ingredients represented by abbreviations (Supplementary Material 2). [file 1116923.f1.zip › 1116923.f1/Supplementary Material(2).docx]

**Supplementary Material:**

Table S2. The names of active ingredients represented by abbreviations

| Herb | Abbreviation | MOLID | Ingredient | PubChem CID |
| --- | --- | --- | --- | --- |
| Du-zhong | DZ2 | MOL002773 | Beta-carotene | 5280489 |
|  | DZ5 | MOL009015 | (-)-Tabernemontanine | 12309360 |
|  | DZ7 | MOL009031 | Cinchonan-9-al,6'methoxy-, (9R)- | 94175 |
|  | DY2 | MOL000422 | Kaempferol | 5280863 |
|  | DY1 | MOL000098 | Quercetin | 5280343 |
|  | DZ3 | MOL007059 | 3-beta-Hydroxymethyllenetanshiquinone | 5318290 |
|  | DZ9 | MOL009055 | Hirsutin_qt | - |
|  | DZ1 | MOL000073 | ent-Epicatechin | 182232 |
|  | DZ6 | MOL009029 | Dehydrodiconiferyl alcohol 4, gamma'-di-O-beta-D-glucopyanoside_qt | - |
|  | DZ8 | MOL009053 | 4-[(2S,3R)-5-[(E)-3-hydroxyprop-1-enyl]-7-methoxy-3-methylol-2,3-dihydrobenzofuran-2-yl]-2-methoxy-phenol | 11824478 |
|  | DZ4 | MOL008240 | (E)-3-[4-[(1R,2R)-2-hydroxy-2-(4-hydroxy-3-methoxy-phenyl)-1-methylol-ethoxy]-3-methoxy-phenyl]acrolein | 21582571 |
|  | DZ10 | MOL011604 | Syringetin | 5281953 |
| Yin-yang-huo | YYH4 | MOL003542 | 8-Isopentenyl-kaempferol | 5318624 |
|  | YYH6 | MOL004380 | C-Homoerythrinan,1,6-didehydro-3,15,16-trimethoxy-, (3.beta.)- | 296195 |
|  | YYH8 | MOL004391 | 8-(3-methylbut-2-enyl)-2-phenyl-chromone | 17861868 |
|  | YYH1 | MOL000006 | Luteolin | 5280445 |
|  | YYH2 | MOL001792 | DFV | 114829 |
|  | YYH3 | MOL003044 | Chryseriol | 5280666 |
|  | YYH5 | MOL004373 | Anhydroicaritin | 5318980 |
|  | YYH7 | MOL004382 | Yinyanghuo A | 5315393 |
|  | YYH9 | MOL004396 | 1,2-bis(4-hydroxy-3-methoxyphenyl)propan-1,3-diol | 12468616 |
| Bu-gu-zhi | BGZ1 | - | Bakuchiol | 5468522 |
|  | BGZ2 | - | Bavachalcone | 6450879 |
|  | BGZ3 | - | Bavachin | 14236566 |
|  | BGZ4 | - | Bavachinin | 10337211 |
|  | BGZ5 | - | Bavachromene | 5321800 |
|  | BGZ6 | - | Bavacoumestan a | 5321811 |
|  | BGZ7 | - | Corylidin | 5316096 |
|  | BGZ8 | - | Corylin | 5316097 |
|  | BGZ9 | - | Corylinal | 44257227 |
|  | BGZ10 | - | Genistein | 5280961 |
|  | BGZ11 | - | Isobavachin | 193679 |
|  | BGZ12 | - | Isoneobavachalcone | 5318608 |
|  | BGZ13 | - | Neobavachalcone | 5320052 |
|  | BGZ14 | - | Neobavaisoflavone | 5320053 |
